# Active hematopoiesis triggers exosomal release of PRDX2 that promotes osteoclast formation

**DOI:** 10.14814/phy2.14745

**Published:** 2021-02-15

**Authors:** Gulzhakhan Sadvakassova, Kerstin Tiedemann, Kieran J. D. Steer, Nicholas Mikolajewicz, Mariya Stavnichuk, Irene In‐Kyung Lee, Zarina Sabirova, Matthias Schranzhofer, Svetlana V. Komarova

**Affiliations:** ^1^ Faculty of Dentistry McGill University Montréal QC Canada; ^2^ Shriners Hospital for Children – Canada Montréal QC Canada; ^3^ Department of Community Health Sciences University of Calgary Calgary Alberta Canada; ^4^ Department of Biomedical Engineering Faculty of Medicine McGill University Montréal QC Canada; ^5^ Lady Davis Institute for Medical Research Jewish General Hospital Montréal QC Canada

**Keywords:** anemia, bone, hematopoiesis, osteoclasts, PRDX2

## Abstract

Hematopoietic disorders, particularly hemolytic anemias, commonly lead to bone loss. We have previously reported that actively proliferating cancer cells stimulate osteoclastogenesis from late precursors in a RANKL‐independent manner. We theorized that cancer cells exploit the physiological role of bone resorption to support expanding hematopoietic bone marrow and examined if hematopoietic cells can trigger osteoclastogenesis. Using phlebotomy‐induced acute anemia in mice, we found strong correlation between augmented erythropoiesis and increased osteoclastogenesis. Conditioned medium (CM) from K562 erythroleukemia cells and primary mouse erythroblasts stimulated osteoclastogenesis when added to RANKL‐primed precursors from mouse bone marrow or RAW264.7 cells. Using immunoblotting and mass spectrometry, PRDX2 was identified as a factor produced by erythroid cells *in vitro* and *in vivo*. PRDX2 was detected in K562‐derived exosomes, and inhibiting exosomal release significantly decreased the osteoclastogenic capacity of K562 CM. Recombinant PRDX2 induced osteoclast formation from RANKL‐primed primary or RAW 264.7 precursors to levels comparable to achieved with continuous RANKL treatment. Thus, increased bone marrow erythropoiesis secondary to anemia leads to upregulation of PRDX2, which is released in the exosomes and acts to induce osteoclast formation. Increased bone resorption by the osteoclasts expands bone marrow cavity, which likely plays a supporting role to increase blood cell production.

## Graphical abstract

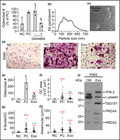

## 1 INTRODUCTION

Anemias lead to bone loss, often severe (Steer et al., 2017), which has been observed in both patients with chronic anemia and replicated in mouse models (Moreau et al., 2012). In chronic hemolytic anemias, erythroid marrow hyperplasia is accompanied by findings of increased bone resorption, including widened medullary space, thinned cortical and trabecular bone, and osteoporosis (Faber et al., 2002; Gurevitch et al., 2007; Moreau et al., 2012; Voskaridou et al., 2006). In keeping, resorption markers correlate with bone loss in patients with β‐thalassemia (Pratelli et al., 2006; Voskaridou et al., 2001; Voskaridou & Terpos, 2004). Other findings also suggest that medullary erythropoiesis stimulates bone resorption, including correlation between degree of bone involvement and the severity of anemia, exclusive involvement of red bone marrow; reversibility of bone changes after anemia resolution (Angelopoulos et al., 2006; Li et al., 2012). It was recently proposed that non‐hemolytic anemias, such as those secondary to nutritional deficiencies, may contribute to bone loss in osteoporosis (Korkmaz et al., 2012; Rutten et al., 2013; Toxqui & Vaquero, 2015). The exact mechanisms underlying anemia‐ induced bone loss is yet incompletely understood.

Numerous studies have demonstrated that all bone cells regulate hematopoiesis (Asada & Katayama, 2014; Compston, 2002; Shiozawa et al., 2008; Teti, 2012), and that hematopoietic cells, in turn, regulate bone remodeling (Malara et al., 2015; Singh et al., 2012; Teti, 2012). Erythropoietin (Epo), the main hormone regulating erythropoiesis, has been suggested to directly contribute to regulation of bone cells (Hiram‐Bab et al., 2015, 2017; Taichman, 2005). The role of key osteoclastogenesis regulators, including Receptor Activator of Nuclear factor κ‐B Ligand (RANKL) and osteoprotegerin (OPG), in mediating the observed bone changes in patients with β‐thalassemia and sickle‐cell anemia has been investigated by several groups. Results show an alteration of the RANKL/OPG axis in thalassemic patients (Angelopoulos et al., 2007; Morabito et al., 2004, 2007; Voskaridou et al., 2006). However, changes in bone mineral density do not correlate with RANKL/OPG levels or ratio (Angelopoulos et al., 2007). Thus, bone changes in patients with hemolytic anemias are consistent with increased osteoclastogenesis by proliferating erythroid cells, which is at least partially RANKL‐independent.

Our previous studies investigating the effect of breast and prostate cancer cells on osteoclast formation and function demonstrated that the ability to actively proliferate was critical for the osteoclastogenic capacity of cancer cells (Guo et al., 2008; Rafiei et al., 2015; Tiedemann et al., 2009). We hypothesize that one of the functions of bone is to accommodate blood cell proliferation and differentiation by stimulating osteoclastic bone resorption to widen the bone marrow space and accommodate for blood cell proliferation and differentiation. We also hypothesize, that this same mechanism may contribute to osteolysis in the pathological conditions associated with marrow hypercellularity, such as tumor growth in cancer metastases to bone. The goal of this study was to examine the interactions between erythropoietic cells and osteoclasts both *in vivo*, using a model of acute anemia induced by phlebotomy (Moreau et al., 2012), and *in vitro*, using K562 erythroleukemia cell line, RAW264.7 monocytic cell line and primary murine erythroblasts and bone marrow osteoclast precursors.

## 2 RESULTS

### 2.1 Acute anemia in mice induces bone marrow erythropoiesis and stimulates osteoclast formation

**FIGURE 1:**
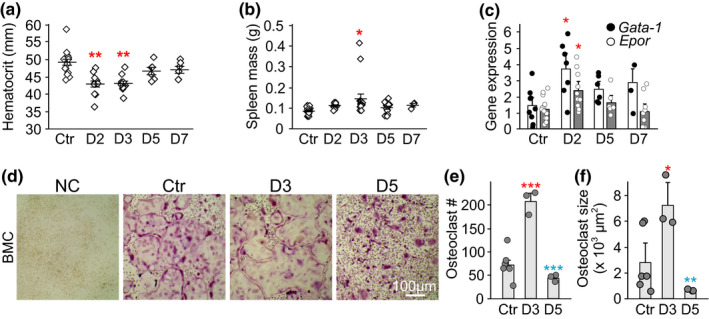
Acute anemia in mice promotes osteoclastogenesis from bone marrow precursors. Acute anemia was induced in female C57BL/6 mice by collecting 10% of total blood volume from saphenous vein. Control mice (Ctr) underwent the same procedure, but no blood was taken. Mice sacrificed at day 2–7 after bleeding. a) Peripheral blood hematocrit, N = 6–16 mice/time point. b) Spleen weight, N = 3–20 mice/time point. c) Gene expression of erythropoietic markers *Gata*‐*1 (black circle)* and *Epor (white circle)* in bone marrow, N = 3–15 mice/time point. d‐f) Bone marrow cells (BMC) were collected from control mice (Ctr) and on day 3 (D3) and day 5 (D5) after induction of anemia, and were cultured for 5 days with MCSF only (50 ng/ml, negative control (NC)), or with MCSF and RANKL (50 ng/ml). d) Representative images of osteoclasts formed in different conditions. Scale bar applies to all images. e) Osteoclast number, N = 3–6 mice/condition. f) Osteoclast size, N = 74–82 osteoclasts/condition from 3–6 mice. Data are means ±SEM, *p < 0.05, **p < 0.01 and ***p < 0.001 by one‐way ANOVA with Tukey post‐test; *red*: compared to control mice, *blue*: compared to D3 mice

To examine if erythropoiesis and osteoclastogenesis are linked *in vivo*, acute anemia was induced in 5–7‐week‐old female C57BL/6 mice by withdrawing about 10% of total blood volume. This procedure resulted in a significant decrease in hematocrit for 3 days, which then steadily increased and reached normal values after 1 week (Figure 1a). Physiologic response to acute blood loss was confirmed by a significant increase in spleen weight (Figure 1b) and induction of hematopoietic transcription factors, *Gata*‐*1* and erythropoietin receptor (*Epor)* in bone marrow (Figure 1c). The maximal changes in erythropoietic transcription factors occurred on day 2–3 post‐bleeding and recovered by day 5. In keeping with this timeline, osteoclast precursors were isolated from the bone marrow of control mice or anemic mice in the active stage (3 days after bleeding) or the recovery stage (5 days after bleeding) and their osteoclastogenic potential was assessed *in vitro*. Compared to control mice, the number and size of osteoclasts formed from precursors of mice in the active stage significantly increased (Figure 1d‐f). In contrast, precursors from mice in the recovery stage demonstrated osteoclastogenesis at the same level as controls (Figure 1d‐f). These data suggest that osteoclastogenesis is promptly regulated by bone marrow hematopoiesis.

### 2.2 Soluble factors produced by erythroleukemia cells promote osteoclast formation

**FIGURE 2:**
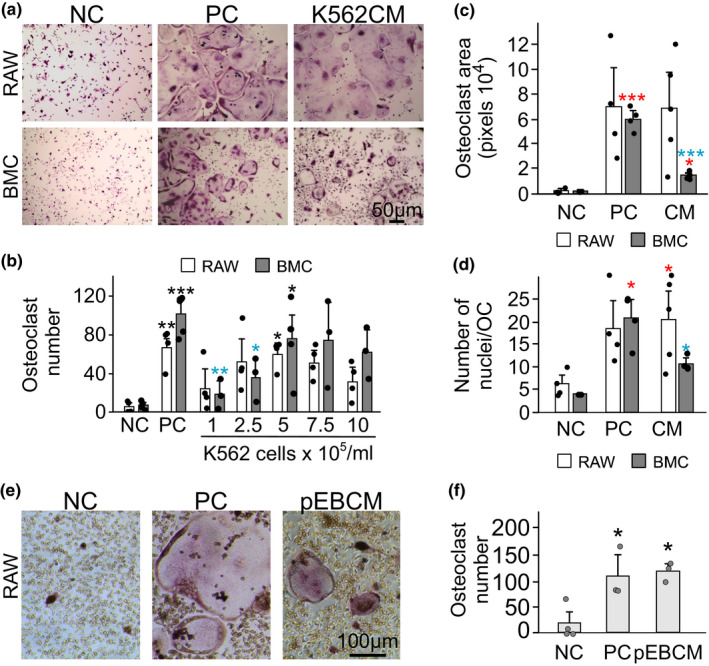
Erythroblastic cells secrete osteoclastogenic factors. a‐d) Conditioned media (CM) were harvested from K562 cells that were plated at 5 x10^5^ cells/ml or in indicated densities (b). RAW 264.7 cells (RAW) were cultured with RANKL (50 ng/ml) for 2 days, or bone marrow cells (BMC) were primed with RANKL and MCSF (50 ng/ml) for 2 days. The osteoclast precursors were washed and cultured for additional 2 days without treatment (negative control, NC), with RANKL (50 ng/ml, positive control, PC) or with 10% CM from K562 cells. a) Representative images of osteoclasts formed in different conditions. Scale bar applies to all images. b) Average osteoclast number. c) Average osteoclast planar area. d) Average number of nuclei per osteoclast. N = 3–5 independent experiments. e‐f) RANKL‐primed RAW 264.7 cells were cultured for 2 days with media conditioned by primary erythroid cells (pEBCM). e) Representative images of osteoclasts formed in different conditions. Scale bar applies to all images. f) Average numbers of osteoclasts, N = 3–6 independent experiments. Data are means ±SEM, *p < 0.05, **p < 0.01 and ***p < 0.001by one‐way ANOVA with Tukey post‐test; *black and red*: compared to negative control, *blue*: compared to positive control

We have previously demonstrated that actively proliferating breast or prostate cancer cells stimulate osteoclast formation from RANKL‐primed precursors (Guo et al., 2008; Rafiei & Komarova, 2013; Tiedemann et al., 2009). We hypothesize that osteoclastogenesis in physiologic states may be similarly regulated by hematopoietic cells residing in the bone marrow. K562 erythroleukemia cells were used as a model for erythroblast proliferation (Andersson et al., 1979; Hoffman et al., 1979). Osteoclast precursors from mouse bone marrow (BMC) were cultured with MCSF and RANKL, or RAW 264.7 monocytes were cultured with RANKL for two days to generate late, RANKL‐primed, osteoclast precursors. Treating RANKL‐primed precursors with conditioned medium (CM) from K562 cells (10%) for 2 days resulted in significant increases in the osteoclast number, size, and nucleation compared to the negative control, which was maintained without RANKL (Figure 2a‐d). In RAW 264.7 cultures, the effect of K562 CM was similar to the positive control, while in K562 CM‐treated BMC cultures osteoclast size and nucleation were lower than in positive control cultures. The effect of K562 CM on osteoclastogenesis strongly depended on the density of K562 cultures (Figure 2b), with the maximal effect achieved by CM from moderately dense (5 × 10^5^ cells/ml) K562 cultures. To confirm the osteoclastogenic effect of erythroblastic cells, media conditioned by primary mouse erythroblasts (pEB) was used to culture RANKL‐primed RAW 264.7 cell (Figure 2e,f). pEB cells that had been differentiating for 24 h significantly increased osteoclast formation when 1% of pEB CM was added to RANKL‐primed RAW 264.7 cells (Figure 2f). Taken together, we conclude that proliferating erythroblastic cells release factors that promote osteoclastogenesis from RANKL‐primed precursors.

### 2.3 Erythropoietin acts as a positive regulator of early osteoclastogenesis

**FIGURE 3:**
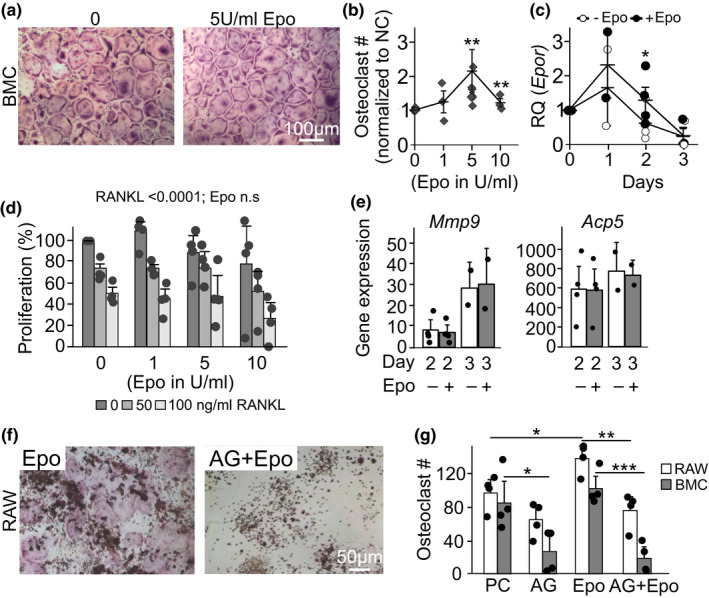
Erythropoietin affects osteoclast formation. RAW 264.7 cells (RAW) were cultured with RANKL (50 ng/ml), or bone marrow cells (BMC) were cultured with MCSF (50 ng/ml) and RANKL (0–100 ng/ml), with or without Epo (0–10 U/ml). a) Representative images of osteoclasts formed in BMC cultures on day 5. Scale bar applies to both images. b) Osteoclasts formed in Epo‐treated BMC cultures were counted and normalized to cultures treated with MCSF and RANKL (100 ng/ml) but without Epo. N = 3–9 independent experiments. c) Relative expression of erythropoietin receptor in BMC treated for 0–3 days with MCSF and RANKL (100 ng/ml) without or with Epo (5 U/ml). N = 2–4 independent experiments. d) Cell proliferation was assessed in BMC cultures treated 2 days with MCSF, RANKL (0–100 ng/ml), and Epo (0–10 U/ml). N = 5 independent experiments. e) Relative expression of *Mmp9 and Acp5* in osteoclast cultures treated with Epo (5 U/ml) for 2–3 days. Data are means ±SD, N = 3 technical replicates. f‐g) RAW 264.7 cells or BMC were cultured for 5 days with RANKL (50 ng/ml, PC, white bars) or RANKL and MCSF (50 ng/ml, PC, gray bars), with or without Epo (Epo, 5 U/ml), Jak2 inhibitor (AG, 5 µM) or a combination thereof (AG+Epo). f) Representative images of osteoclasts formed in RAW cultures on day 5. Scale bar applies to both images. g) Average numbers of RANKL‐treated osteoclasts from RAW and RANKL and MCSF‐treated BMC cells formed with or without Epo (Epo, 5 U/ml), Jak2 inhibitor (AG, 5 µM) or a combination (AG+Epo). N = 4 independent experiments. Data are means ±SEM, *p < 0.05, **p < 0.01 and ***p < 0.001 compared to control without Epo by one‐way ANOVA (b, c), two‐way ANOVA (d) or Student's t‐test (e, g).

Since Epo was suggested to directly regulate osteoclast formation (Hiram‐Bab et al., 2017), we next examined its potential involvement. When bone marrow precursors were cultured with MCSF and RANKL, or RAW 264.7 cells were cultured with RANKL and Epo (5–10 U/ml) for 5 days, osteoclastogenesis was significantly augmented at intermediate Epo levels (Figure 3a‐b). *Epor* was expressed in osteoclast precursors; however, its expression progressively decreased as osteoclasts were formed, although with a delay in cultures treated with Epo (Figure 3c). Neither the proliferation of osteoclast precursors (Figure 3d) nor the osteoclastogenic markers of gene expression (Figure 3e) was significantly affected by Epo. We assessed the role of JAK2 signaling pathway, which is activated by Epo (Shiozawa et al., 2010). While pharmacological inhibition of JAK2‐STAT3 signaling significantly reduced Epo‐stimulated osteoclastogenesis, it also dramatically reduced osteoclast formation in the positive control cultures (Figure 3f). These data suggest that Epo acts as a positive regulator of osteoclastogenesis; however, its action is likely restricted to early osteoclast precursors that still express Epo receptor.

### 2.4 Acute anemia leads to an increase in bone marrow peroxiredoxin‐2

**FIGURE 4:**
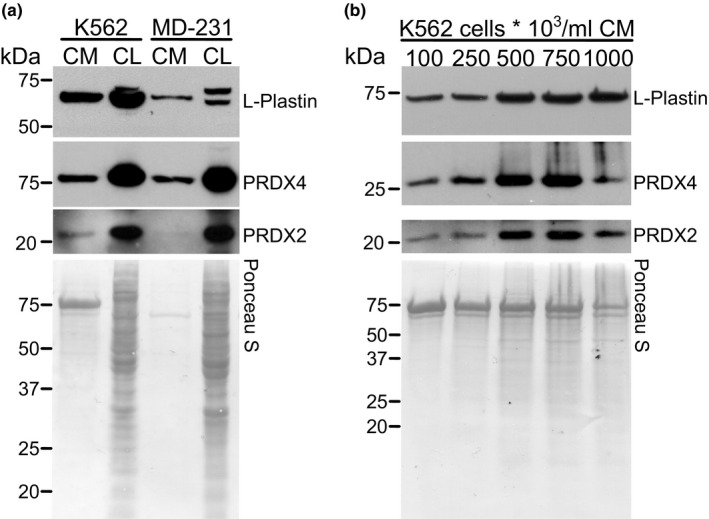
Identification of L‐plastin and PRDXs in K562 cells. Conditioned media (CM) was collected and TCA precipitated, and the protein was extracted from cell lysates (CL) of K562 and MDA‐MB‐231 cells (MD‐231). a) Intracellular and released L‐plastin, PRDX2 and PRDX4 in K562 and MDA‐MB‐231 cells were assessed by immunoblotting. b) K562 cells were cultured at different cell densities (10^5^–10^6^ cells/ml) and the levels of L‐plastin, PRDX2 and PRDX4 in CM were assessed by immunoblotting. Ponceau stain (lower gels) was used as loading control. Shown are representative immunoblots from one out five independent experiments.

**FIGURE 5:**
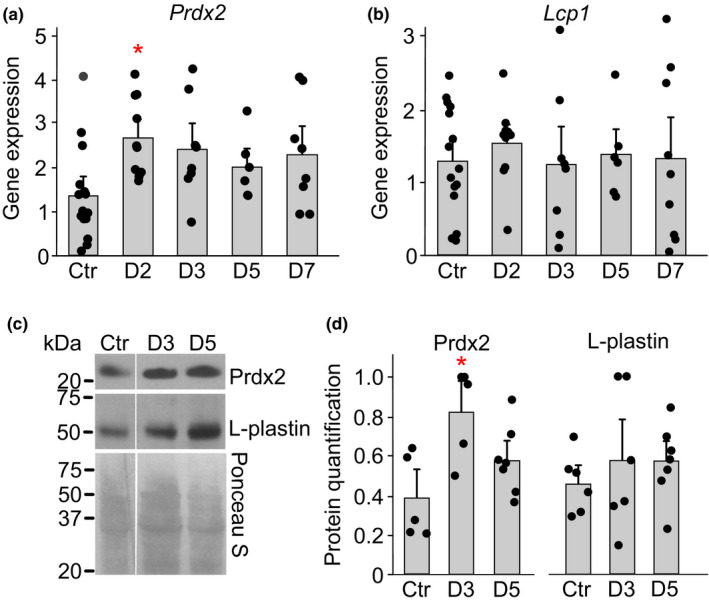
Acute anemia in mice increase the level of Prdx2 in bone marrow. Acute anemia was induced in female C57BL/6 mice by collecting 10% of total blood volume from saphenous vein, after which groups of animals were sacrificed at days 2, 3, 5 and 7. a‐b) RNA was isolated from bone marrow and gene expression for *Prdx2* and *Lcp1* was assessed. c‐d) Protein levels of Prdx2 and Lcp1 in bone marrow were assessed by immunoblotting. c) Representative immunoblots for Prdx2 (*top*), Lcp1 (*middle*) and Ponceau S‐stained gel (*bottom*) used for normalization. d) Average protein expression of Prdx2 and L‐plastin. For each immunoblot, protein signal was first normalized to ponceau staining, and then to the maximum signal within the immunoblot. Data are means ±SEM, N =  5–15 mice per condition, *p < 0.05 by one‐way ANOVA with Bonferroni post‐test, *red*: compared to control

We have previously established that osteoclastogenesis can be induced by both antioxidant enzyme peroxiredoxin‐4 (PRDX4) (Rafiei et al., 2015) and L‐plastin (Tiedemann et al., 2019), which is an actin‐bundling protein secreted by human breast and prostate cancer cells. Similar to MDA‐MB‐231 breast cancer cells, K562 cells produced and released L‐plastin and PRDX4 (Figure 4a). Furthermore, these proteins were secreted in a cell‐density dependent manner from K562 cells with a maximum at the intermediate density range (Figure 4b). Using mass spectrometry, 80 peptides were identified for L‐plastin, as well as 22 for PRDX4 in K562 CM. Of interest, another member of peroxiredoxin family, PRDX2, was identified by mass spectrometry with a total of 24 peptides. PRDX2 was produced and secreted in a cell density‐dependent manner by K562, but not MDA‐MB‐231 cells (Figure 4a, b). In mice with acute anemia, gene and protein expression of PRDX2 was significantly increased in the bone marrow 2–3 days after bleeding (Figure 5a). In contrast, gene expression for L‐plastin (*Lcp1*) was unaffected (Figure 5b). Of note, though some mice had dramatic increase in L‐plastin protein expression (Figure 5b), L‐plastin protein levels were not significantly affected by acute anemia on average (Figure 5c‐d). These data suggest that Prdx2 is the candidate mediator of osteoclastogenesis induced by hematopoietic cells.

### 2.5 Exosomal release by K562 cells induces osteoclast differentiation

**FIGURE 6:**
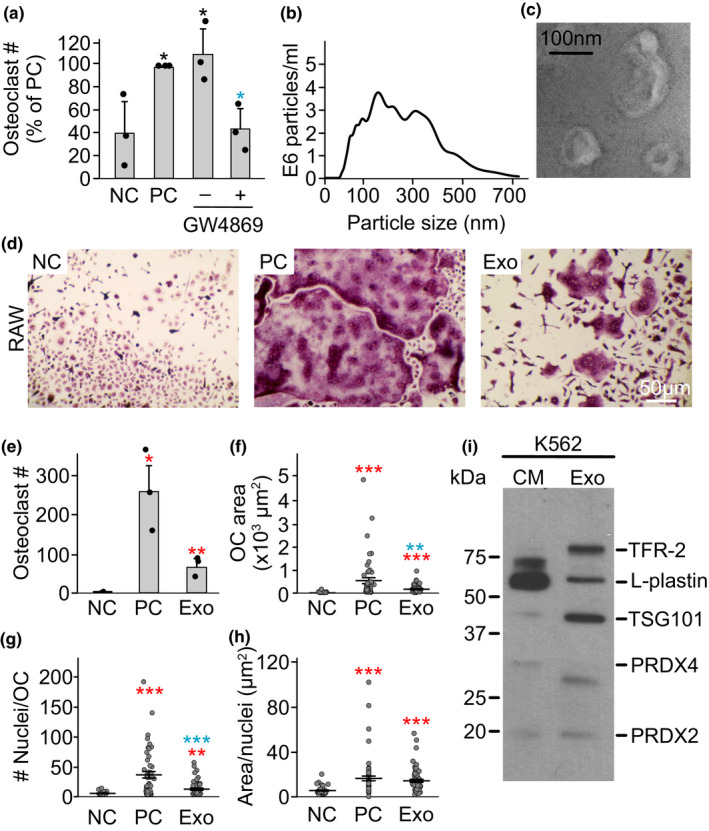
Exosomes from K562 cells induce osteoclast differentiation. a) RAW 264.7 cells were primed with RANKL (50 ng/ml) for 2 days and then cultured for an additional 2 days without RANKL treatment (negative control, NC), with RANKL (50 ng/ml, positive control, PC) or 10% CM from K562 cells +/− exosome inhibitor GW4869 (10 µM). N  =  3. b) K562 cells were cultured for 24 h then exosomes were purified, and the distribution of particle sizes was analyzed by Nano sight. c) Representative transmission electron microscopy image of exosomes purified from K562 CM. d‐h) RAW 264.7 cells were primed with RANKL (50 ng/ml) for 2 days and then cultured for an additional 2 days without RANKL treatment (negative control, NC), with RANKL (50 ng/ml, positive control, PC) or with purified exosomes (Exo, 10 µl) from K562 cells. d) Representative images of TRAP‐stained osteoclasts formed in RAW cultures on day 5. Scale bar applies to all images. e) Average osteoclast number; f) average area per osteoclast; g) number of nuclei per osteoclast; h) average area/nucleus. N = 37–50 osteoclasts/condition; i) Immunoblotting for exosomal markers TFR‐2 and TSG101, L‐plastin, PRDX4 and PRDX2 in K562 CM and purified exosomes (Exo). Data are means ±SEM, *p < 0.05, **p < 0.01 and ***p < 0.001 assessed by Student's t‐test; *red*: compared to negative control, *blue*: compared to ‐GW4869 or positive control.

We have previously shown that osteoclastogenic factors are released from breast cancer cells in exosomes (Tiedemann et al., 2019). Pharmacological inhibition of exosome generation by GW4869 in K562 cells significantly reduced their osteclastogenic potential (Figure 6a). Exosomes isolated from K562 cells using differential centrifugation had an average vesicular size of 147 nm (Figure 6b), and a characteristic circular appearance with a central depression on transmission electron microscopy (Figure 6c). The addition of the purified K562 exosomal fraction to osteoclast precursors promoted osteoclast formation compared to the negative control, even though the resulting osteoclasts were smaller than those generated in positive control cultures (Figure 6d‐h). Immunoblotting of K562‐derived exosomes demonstrated the presence of exosomal markers, transferrin receptor‐2 (TFR‐2) and TSG 101, as well as L‐plastin and PRDX2 (Figure 6i). In contrast, PRDX4 was present in conditioned medium, but the corresponding band in the exosomes was of reduced molecular weight, potentially indicating degradation. Thus, osteoclastogenic factors, including PRDX2, are released from hematopoietic cells at least in part with exosomes.

### 2.6 Induction of osteoclast formation by recombinant human peroxiredoxin‐2

**FIGURE 7:**
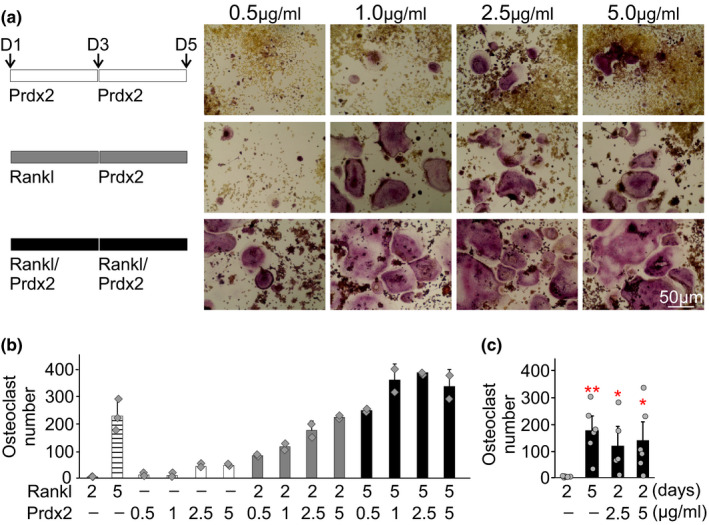
Recombinant Prdx2 induces osteoclast differentiation from RANKL‐primed precursors. RAW 264.7 cells were cultured for 4 days without RANKL (white bars), were treated with RANKL (50 ng/ml) for 4 days (black bars) or were RANKL‐primed (treated with RANKL 50 ng/ml for 2 days and cultured for an additional 2 days without RANKL, grey bars). a,b) Prdx2 at concentrations 0.5, 1, 2.5, or 5 µg/ml was added to untreated, RANKL‐treated and RANKL‐primed RAW 264.7. Shown are representative images of TRAP‐positive osteoclasts (a) and average number of osteoclasts formed in different conditions on day 4 (b). Scale bar applies to all images. Data are means ±SD, N  =  2–3 replicates. c) Average number of osteoclasts formed when recombinant PRDX2 (2.5 or 5 µg/ml) was added to RANKL‐primed osteoclast precursors. Data are means ±SEM, N = 5–6 independent experiments, *p < 0.05 and **p < 0.01 compared to RANKL‐primed cultures by Student's t‐test.

We next examined if PRDX2 can stimulate osteoclast formation similar to PRDX4 (Rafiei et al., 2015) and L‐plastin (Tiedemann et al., 2019). When added to untreated RAW 246.7 osteoclast precursors, recombinant PRDX2 was only able to minimally stimulate osteoclast formation (Figure 7a, b; *open bars*). When PRDX2 was added together with RANKL for the full culture duration, it did not augment osteoclastogenesis compared to the positive control (RANKL‐treated RAW 246.7 cells; *lined bars*) (Figure 7a, b; *black bars*). However, when added to RANKL‐primed osteoclast precursors, PRDX2 dose‐dependently stimulated osteoclastogenesis (Figure 7a, b; *grey bars*). PRDX2 significantly stimulated osteoclast formation compared to the negative control (late osteoclast precursors cultured for 2 days with RANKL) to the levels observed in the positive control cultures (Figure 7c). Thus, PRDX2 was able to substitute for RANKL and stimulate osteoclast formation from late osteoclast precursors.

## 3 DISCUSSION

In this study, we demonstrate that acute anemia is associated with stimulation of osteoclastogenesis and that actively proliferating erythroblasts produce osteoclast‐stimulating factors which is distinct from RANKL or Epo. We found that the osteoclastogenic factors previously identified as important for cancer‐induced osteolysis, L‐plastin and PRDX4, are released from erythropoietic cells. In addition, we demonstrate that PRDX2 was produced by erythroblastic cells, released in exosomes, and significantly increased *in vivo* following phlebotomy. Importantly, application of recombinant PRDX2 to late osteoclast precursors directly stimulated osteoclast formation. This study identifies PRDX2 as a novel regulator of osteoclastogenesis with a potential role in adaptive osteolysis regulated by hematopoietic bone marrow.

Our results showed that actively proliferating hematopoietic cells stimulate osteoclastogenesis both *in vitro* and *in vivo*. A strong link between hematopoiesis and bone health has been long noticed (Teti, 2012). We have previously shown that the degree of bone loss is proportional to the degree of bone marrow hematopoietic cellularity in hematological disorders of bone marrow origin. Chronic hemolytic anemias in particular are associated with a significant bone loss (Steer et al., 2017). Acute anemia caused by bleeding or hemolysis leads to increased bone marrow erythropoiesis (Eymard et al., 2015). In mice, anemia induced by phlebotomy, phenylhydrazine, or plasmodium infection lead to osteoclast activation; however, bone loss was observed only in the models with hemolysis (Moreau et al., 2012). This difference is likely due to the relatively fast recovery of hematopoietic parameters after acute bleeding since bone loss was reported in continuously bled mice (Gurevitch & Slavin, 2006). Stimulation of bone marrow hematopoiesis in the absence of anemia similarly was shown to result in bone loss (Frisch et al., 2009). Consistently, our findings demonstrate that osteoclastogenic potential of bone marrow derived precursors transiently increases following anemia induced by bleeding. The limitations of our study were that we only assessed the impact of anemia in female mice, and that long‐term impact of anemia on bone health was not possible to assess because of the transient nature of phlebotomy‐induced anemia. Nevertheless, these data suggest that osteoclastogenesis is stimulated by local, rather than systemic factors associated with bone marrow hematopoiesis.

Erythropoietin, the main hormone that stimulates erythrocyte production, has been suggested to directly and indirectly regulate bone cells (Hiram‐Bab et al., 2017; McGee et al., 2012). Epo receptor (Epor) was found on osteoblasts (Balaian et al., 2018; Suresh et al., 2020), and, consistent with previous studies (Shiozawa et al., 2010), we demonstrate that Epor is present on osteoclast precursors but is downregulated as they differentiate into osteoclasts. Epo was previously reported to directly stimulate osteoclast formation *in vitro* (Hiram‐Bab et al., 2015), which is consistent with our observations. However, our data suggest that the effects of Epo are likely limited to early stages of osteoclastogenesis, while additional factors released from hematopoietic cells act on late osteoclast precursors. Exogenous Epo administration in mice was shown to stimulate bone resorption (Singbrant et al., 2011); however, these experiments cannot distinguish the direct effects of Epo on bone cells and the effects mediated by hematopoietic cells stimulated by the exogenous Epo. While Epo is an important regulator of bone health, we propose that it likely acts synergistically with local factors produced by actively proliferating erythroblasts.

We identified PRDX2 as a potential osteoclastogenic factor released from hematopoietic cells. PRDX2 is an abundant cytosolic protein in RBCs that plays an essential role in protection from oxidative stress during heme oxidation (Johnson et al., 2005; Lee et al., 2003; Lee, 2020; Nagababu et al., 2013). PRDX2 has been shown to stabilize hemoglobin and prevent hemolysis during oxidative stress (Han et al., 2012). PRDX2‐deficient mice exhibit hemolytic anemia associated with ineffective erythropoiesis and oxidative DNA damage (Kwon et al., 2012; Matte et al., 2015). Importantly, PRDX2‐deficient mice were also shown to have higher levels of bone mass than those of wild‐type mice (Kim et al., 2019), which has been linked to the effects of PRDX2 on BMP2‐induced osteoblast differentiation. Our study suggests that this lack of hemolytic anemia‐associated bone loss may also be due to the absence of PRDX2‐mediated osteoclastogenesis. We demonstrate that PRDX2, normally a cytoplasmic protein, is released from hematopoietic cells at least in part with exosomes. This route of release is similar to that of cancer‐released factors, including L‐plastin (Tiedemann et al., 2019), which is also released by hematopoietic cells but was not consistently associated with osteoclastogenesis. Most importantly, we demonstrate that the addition of recombinant PRDX2 to late osteoclast precursors directly and independently of RANKL stimulated the formation of multinucleated osteoclasts. These data suggest that, like cancer cells, hematopoietic cells release biologically active exosomes, and PRDX2 is one of the factors that stimulates osteoclastogenesis in response to anemia.

The adaptive interactions between bone and bone marrow are important for the function of both compartments in physiological conditions. Bone marrow provides precursors for successful bone remodeling, and bone resorption provides physical space to accommodate hematopoietic cell proliferation and differentiation. Pathological bone destruction in diseases associated with hypercellular bone marrow, such as hemolytic anemias, hematologic neoplasia, inflammatory conditions, or metastatic bone disease likely result from abnormal or extreme function of this physiologic system. The identification of the mechanisms underlying these interactions, such as release of osteoclastogenic factors PRDX2, PRDX4 and L‐plastin by proliferating cells, may lead to the development of novel targeted therapeutic interventions for inflammatory arthritis, metastatic bone disease and hematolytic anemias.

## 4 MATERIAL AND METHODS

### 4.1 Cell cultures

Mouse bone marrow cells were collected as previously described (Armstrong et al., 2009). This study was carried out in accordance with the recommendations of the Canadian Council on Animal Care. The protocol was approved by the McGill University Animal Care Committee. Mouse bone marrow cells were collected from 6 weeks old C57BL6/J mice (Charles River). Cells were cultured in 75 cm^2^ tissue culture flasks (1.5 × 10^7^ cells per flask) with human recombinant macrophage‐colony stimulating factor (M‐CSF, 25 ng/ml, 300–25, PeproTech Inc.) for 24 h, then non‐adherent cells were collected and plated at 5 × 10^4^ cells/cm^2^ in α‐MEM medium supplemented with 100 units/ml penicillin, 100 µg/ml, streptomycin and 10% fetal bovine serum, M‐CSF (50 ng/ml) and recombinant GST‐RANKL (50 ng/ml). Medium was changed every other day. On day 5 cell cultures were fixed using 10% formalin (23‐245‐685, Fisher) and stained for tartrate‐resistant acid phosphatase (TRAP, Sigma‐Aldrich Co, 387A). Osteoclasts were identified as multinucleated (>3 nuclei) TRAP‐positive cells and were further characterized by image analysis using PixeLINK Capture SE® software (PixeLINK) and Image J. The MDA‐MB‐231 breast cancer cell line was provided by Dr. Peter Siegel (McGill University, Montreal) and cultured as previously described (Tiedemann et al., 2009). K‐562 cells (CCL‐243™, American Type Culture Collection ‐ ATCC) and RAW 264.7 cells (TIB‐71™, American Type Culture Collection ‐ ATCC) were cultured in DMEM supplemented with L‐glutamine, 1 mm pyruvate, 100 units/ml penicillin, 100 µg/ml, streptomycin, and 10% FBS. *i*) RAW 264.7 cells were plated at 5 × 10^3^ cells/cm^2^, and 24 h later (day 1) recombinant GST‐RANKL (50 ng/ml) was added. On days 2–3, cells were supplemented with fresh media with or without RANKL (50 ng/ml) or K562 CM (10%) with or without erythropoietin (5 U/ml, cultured for additional 2 days, fixed, and stained for TRAP. *ii*) RAW 264.7 cells were plated at 5 × 10^3^ cells/cm^2^, and 24 h later, recombinant Prdx2 (MyBioSource, MBS1399869) at concentrations 0.5, 1, 2.5, or 5 µg/ml, was added to untreated, RANKL‐treated and RANKL‐primed RAW 264.7. At day 5 the cells were fixed in formalin and stained with TRAP.

Primary erythroid (pEB) cells were cultured as described (Dolznig et al., 2001; Lindern et al., 2001; Schranzhofer et al., 2006). Briefly, cells were grown from fetal livers that were obtained from E12.5 embryos of wild‐type (FVB.129[B6] background) mice and resuspended in serum‐free StemPro‐34 medium plus Nutrient Supplement (Invitrogen‐Gibco, Carlsbad, USA) plus 2 U/mL human recombinant erythropoietin (Epo; 100 ng/ml), murine recombinant stem cell factor (SCF; 100 ng/ml), the synthetic glucocorticoid dexamethasone (Dex; 10 µm), and insulin‐like growth factor 1 (IGF‐1; 40 ng/ml).

### 4.2 In vivo studies

Animal studies were conducted in compliance with McGill University guidelines established by the Canadian Council on Animal Care. Female C57BL/6 mice (5–7 weeks old, Charles River) were used for anemia model experiments (Moreau et al., 2012). To induce anemia in the mice, 10% of the circulating blood volume (63–80 ml/kg) was safely removed once from the saphenous vein (Diehl et al., 2001). To avoid coagulation of blood 6 µl of 6% EDTA in PBS was added to the collection tubes. Mice in the control group were poked with the needle. At the end of the experiment (day 2, 3, 5 and 7) blood and spleen were for collected for evaluation of hematocrit and spleen weight; and tibia and femur were collected for extraction of bone marrow for osteoclastogenesis and gene expression analysis.

### 4.3 Cell culture reagents

Fetal bovine serum (FBS) was from HyClone (SH 30396‐03), Dulbecco's modified Eagle's medium (DMEM), Alpha MEM (αMEM, 310‐022‐CL), sodium pyruvate (600‐110‐EL), L‐glutamine (609‐065‐EL), penicillin/streptomycin (450‐201‐EL), trypsin/ethylenediaminetetraacetic acid (T/E, 325‐042‐EL) were from Wisent Inc. Recombinant human M‐CSF (300‐25) was from Peprotech Inc. Recombinant glutathione S‐transferase‐soluble RANKL (GST‐RANKL) was purified from the clones kindly provided by Dr. M.F. Manolson (University of Toronto).

### 4.4 Preparation of conditioned medium

K562 cells were cultured at different densities (1–10 × 10^5^ cells/ml) and parental MDA‐MB‐231 cells were cultured in 75‐cm^2^ flasks to 80% confluence and rinsed twice with PBS, 10 ml of serum free medium was added, and cells were cultured for additional 24 h. The conditioned medium was collected and centrifuged (100 × g, 5 min), and then the supernatant was filtered (0.2‐μm filter) and stored (−80°C).

### 4.5 Cell proliferation

Bone marrow cells were plated at a density of 25,000 cells/cm^2^ (96‐well). The following day the medium was replenished with serum‐free medium for 2 hr, followed by the addition of erythropoietin (0–10 ng/ml) and RANKL (0–100 ng/ml). At day 3 medium was changed and the cells were incubated with 10% of Alamar blue (Thermofisher, Y00‐025), after 4 h of incubation the absorbance was read at 570 nm (Rampersad, 2012).

### 4.6 Mass Spectrometry

Proteins from K562 CM were precipitated with 55% trichloroacetic acid (25% from CM volume) and 1% Triton X‐100 (14% from CM), centrifuged at 13,000 x g for 10 min. Protein precipitate from 1.5 ml of CM was washed with 700 µl of ice‐cold acetone. 60 µl loading buffer was added to the precipitate and used for loading on 2 parallel lines on a 7–15% SDS‐PAGE gel followed by Coomassie‐blue staining and immunoblotting with anti‐PRDX2. The band corresponding to PRDX2 on western blot was excised from the Coomassie‐blue stained gel and reduced with DTT, alkylated with iodoacetic acid, and was digested with trypsin. The lyophilized peptides were re‐solubilized in 0.1% aqueous formic acid/2% acetonitrile, the peptides were loaded onto a Thermo Acclaim Pepmap (Thermo, 75 µM ID X 2 cm C18 3 µm beads) precolumn and then onto an Acclaim Pepmap Easyspray (Thermo, 75 µm X 15 cm with 2 µm C18 beads) analytical column separated using a Dionex Ultimate 3000 µHPLC at 220 nl/min with a gradient of 2–35% organic (0.1% formic acid in acetonitrile) over 2 h. Peptides were analyzed using a Thermo Orbitrap Fusion mass spectrometer operating at 120,000 resolutions (FWHM in MS1, 15,000 for MS/MS) with HCD sequencing all peptides with a charge of 2+ or greater. The raw data were converted into *.mgf format (Mascot generic format) searched using Mascot 2.3 against human sequences (Swissprot). The database search results were loaded onto Scaffold Q+ Scaffold_4.4.8 (Proteome Sciences) for spectral counting, statistical treatment and data visualization.

### 4.7 Isolation of exosomes

Exosomes were purified from the K562 CM according to the procedure by Vlassov and colleagues (Vlassov et al., 2012). Briefly, cells were cultured until they reached the exponential growth phase. 500,000–700,000 cells/ml were washed and plated in serum‐free medium. After 24 h, 80–90 ml of CM was collected and centrifuged 100 × g for 10 min to remove cell debris. The supernatant was further centrifuged at 2,500 × g for 15 min and then filtered by a 0.22 µm filter (Millipore) before final ultracentrifugation at 100,000 × g for 2 h. The supernatant was discarded, and the exosome pellet was washed twice with PBS and intermittent ultracentrifugation at 100,000 × g for 2 h. 100 µl of PBS was added to exosomes, mixed well and added to osteoclast precursors.

### 4.8 Nanoparticle Tracking Analysis

Extracellular vesicle profiling entailed measurements of size and numbers of particles secreted from cells cultured in serum depleted medium using the NS500 nanoparticle tracking analysis system (Nanosight, Amesbury, UK) as described previously (Dragovic et al., 2011).

### 4.9 Test Compounds

Before use, the neutral sphingomyelinase inhibitor GW4869 (used to inhibit exosome production) was resuspended in DMSO (7.5 mm) and aliquoted and stored at −70°C. Upon use, GW4869 was solubilized in 1/20 volume of 5% methanesulfonic acid (Trajkovic et al., 2008) (working solution 10 μm). Erythropoietin (Eprex, 20000 U/ml, Rexall), AG490 (JAK2 inhibitor, Cedarlane, 100594480) was added to osteoclast cell cultures on day 2–3, incubated for additional 2 days before fixation and staining with TRAP.

### 4.10 Protein Extraction and Immunoblotting

Cell lysates were extracted in RIPA lysis buffer containing 50 mM Tris, pH 7.4, 150 mM NaCl, 1% Nonidet P‐40, 1 mM EDTA, 1 mg/ml aprotinin, 2 mg/ml leupeptin, 0.1 mM phenylmethylsulfonyl fluoride, 20 mM sodium fluoride, 0.5 mM sodium orthovanadate and centrifuged at 12,000 × g for 10 min at 4°C. The supernatant was collected, and protein was measured using a Quant‐iT™ protein assay kit (Invitrogen). 50–100 µg of lysates were separated on a 10% SDS‐PAGE and transferred to a nitrocellulose membrane (0.45 µm, 162–0115, Bio‐Rad) using 10 mM sodium borate buffer. Transfer efficiency was determined using Ponceau S (T9026, Sigma). The membranes were blocked in 5% milk for 1 h at room temperature followed by overnight incubation at 4°C with primary antibodies: anti‐L‐plastin (1:300, MA5‐11921, Thermo Fisher), anti‐PRDX2 (1:200, AB109367, Abcam or MBS2010800, MyBiosource), anti‐PRDX4 (1:200, AB15574, Abcam), anti‐TFR‐2 (B‐6) (1:100, Santa Cruz, SC‐376278), α‐tubulin (1:5000, T9026, Sigma), anti‐TSG (1:150, Ab83, Cedarlane). The blots were washed, incubated with horseradish peroxidase‐conjugated secondary antibodies (1:1500, anti‐mouse, 170‐5047; 1:1500, anti‐rabbit, 170‐5046; Bio‐Rad) and visualized with a chemiluminescence system (Super signal West Pico; 34080, Pierce).

### 4.11 Transmission Electron Microscopy

Purified exosomes were washed by ultracentrifugation (100,000 × g, 90 min, 4°C) in 0.2 M hydroxyethyl piperazineethanesulfonic acid (HEPES) pH 7.25 solution. All supernatant was removed, and the pellet was resuspended in 2.5% glutaraldehyde in 0.1 M HEPES pH 7.25 solution. 5 µl of fixed exosome solutions were absorbed on each glow‐discharged carbon‐coated nickel grid and allowed to air dry for 5 minutes. Uranyl formate solutions were prepared by dissolving 7.5 µg uranyl formate in 500 µl boiling water and subsequently adding 1.25 µl 10 N NaOH and vigorous shaking. Solutions were then centrifuged at 21,000 × g for 5 minutes to remove debris and supernatants were collected and used. The grids were negatively stained with 5 µl of the prepared uranyl formate solution and allowed to air‐dry for 15 min. Exosomes were imaged using a Philips TECNAI 12 electron transmission microscope. Microscope tension was set to 120 kV and grid was inserted to the sample holder. After ensuring vacuum was maintained, the height of the beam was adjusted using the wobbler function. Intensity, focus, and magnification were adjusted accordingly, and AMT camera was used to capture images.

### 4.12 Quantitative real‐time PCR

Total RNA was isolated from primary cultures (isolated from mouse tibia and femur), using the RNeasy mini kit and QIAshredder columns (Qiagen, Cat. No. 74104 and 79654). For real‐time PCR, 1 μg of total RNA was reverse transcribed using a high capacity cDNA reverse transcription kit (Applied Biosystems, 4368813). Real‐time PCR was performed using 7500 Applied Biosystems instrument using TaqMan probes with the universal PCR Master Mix (Life Technologies, Cat. No. 4304437) in a total volume of 20 µl. The following Taqman probes were used: L‐plastin, (*Lcp1*, Mm01310735_m1); Peroxiredoxin 2, (*Prdx2*, Mm04208213_m1); Matrix metalloprotease 9, (*Mmp9*, Mm00600163_m1); Trap (*Acp5*, Mm00475698_m1); erythroid transcription factor (*Gata*‐*1*; Mm00484679_g1); erythropoietin receptor (*Epor*, Mm00438760_m1) and glyceraldehyde 3‐phosphate dehydrogenase (*Gapdh*, Mm99999915_g1).

### 4.13 Statistics

Data are presented as means ±standard error of the mean (SEM) with sample size (N) indicating the number of independent experiments, or as means ±standard deviation (SD) with sample size (N) indicating the number of samples. Differences were assessed by ANOVA or Student's t‐test and accepted as statistically significant at *p* < 0.05.

## CONFLICTS OF INTEREST

The authors declare no conflict of interest.

## AUTHORS CONTRIBUTION

The conception and design of the study: GS, KT, SVK. The acquisition of data: GS, KT, NM, MS, ZS, KJDS, II‐KL. Analysis and interpretation of data: GS, KT, NM, MS, ZS, KJDS, SVK. Drafting the article: GS, KT, SVK. Culturing and acquiring CM from primary erythroid cells: MSH. Critical revision: GS, KT, MS, NM, KJDS, SVK. Approval of the final version: All co‐authors.
